# Substance Abuse and Cognitive Decline: The Critical Role of Tau Protein as a Potential Biomarker

**DOI:** 10.3390/ijms26157638

**Published:** 2025-08-07

**Authors:** Liliana Rebolledo-Pérez, Jorge Hernández-Bello, Alicia Martínez-Ramos, Rolando Castañeda-Arellano, David Fernández-Quezada, Flavio Sandoval-García, Irene Guadalupe Aguilar-García

**Affiliations:** 1 Departamento de Biología Molecular y Genética, Centro Universitario de Ciencias de la Salud (CUCS), Universidad de Guadalajara, Guadalajara 44100, Mexico; liliana.rebolledo9928@alumnos.udg.mx (L.R.-P.); jorge.hernandezbello@cucs.udg.mx (J.H.-B.); 2 Departamento de Neurociencias, Centro Universitario de Ciencias de la Salud, Universidad de Guadalajara, Guadalajara 44100, Mexico; alicia.mramos@academicos.udg.mx (A.M.-R.); david.fernandez@academicos.udg.mx (D.F.-Q.); flavio.sandoval@academicos.udg.mx (F.S.-G.); 3 Departamento de Ciencias Biomédicas, Centro Universitario de Tónala, Universidad de Guadalajara, Guadalajara 44100, Mexico; rolando.castaneda@academicos.udg.mx

**Keywords:** Tau protein, cognitive dysfunction, substance abuse, neurodegeneration, p-Tau

## Abstract

Tau protein is essential for the structural stability of neurons, particularly through its role in microtubule assembly and axonal transport. However, when abnormally hyperphosphorylated or cleaved, Tau can aggregate into insoluble forms that disrupt neuronal function, contributing to the pathogenesis of neurodegenerative diseases such as Alzheimer’s disease (AD). Emerging evidence suggests that similar Tau-related alterations may occur in individuals with chronic exposure to psychoactive substances. This review compiles experimental, clinical, and postmortem findings that collectively indicate a substance-specific influence on Tau dynamics. Alcohol and opioids, for instance, promote Tau hyperphosphorylation and fragmentation through the activation of kinases such as GSK-3β and CDK5, as well as proteases like caspase-3, leading to neuroinflammation and microglial activation. Stimulants and dissociatives disrupt insulin signaling, increase oxidative stress, and impair endosomal trafficking, all of which can exacerbate Tau pathology. In contrast, cannabinoids and psychedelics may exert protective effects by modulating kinase activity, reducing inflammation, or enhancing neuroplasticity. Psychedelic compounds such as psilocybin and harmine have been demonstrated to decrease Tau phosphorylation and facilitate cognitive restoration in animal models. Although the molecular mechanisms differ across substances, Tau consistently emerges as a convergent target altered in substance-related cognitive disorders. Understanding these pathways may provide not only mechanistic insights into drug-induced neurotoxicity but also identify Tau as a valuable biomarker and potential therapeutic target for the prevention or treatment of cognitive decline associated with substance use.

## 1. Introduction

Substance use and substance use disorders (SUDs) represent a major global health burden. Alcohol remains the most widely consumed psychoactive substance, with approximately 2.3 billion users worldwide [1,2]. Furthermore, in 2019, an estimated 275 million people used substances other than alcohol and tobacco, predominantly adolescents and young adults. Notably, cannabis was the most used illicit drug, with around 200 million users and a significant contribution to drug-related offenses. In contrast, opioids were associated with the highest number of drug-related deaths, which have increased by 41% over the past decade [3,4].

For this review, “substance abuse” refers to both chronic use and clinically diagnosed SUDs, as defined by the DSM-5 [5]. We focus on the most studied categories of psychoactive substances with available data regarding their impact on Tau protein: (1) depressants (e.g., alcohol), (2) stimulants (e.g., cocaine, methamphetamine), (3) opioids (e.g., heroin, morphine), (4) cannabinoids (e.g., THC, CBD), (5) dissociatives (e.g., ketamine), and (6) psychedelics (e.g., psilocybin, LSD, harmine). These categories were selected based on pharmacological classifications and the existing literature linking them to Tau-related neuropathology.

The intricate interplay between chronic substance abuse and cognitive dysfunction presents a substantial challenge in the domains of neuroscience and addiction medicine. Chronic substance abuse, particularly, has been consistently linked to cognitive impairments, which are attributed to a range of neurobiological alterations within the brain [6]. Among the molecular targets affected by these substances, Tau protein has gained attention due to its central role in cytoskeletal integrity and neuronal plasticity.

Tau protein, which is primarily linked to AD, becomes hyperphosphorylated in individuals who abuse substances. This hyperphosphorylation leads to microtubule disintegration and the formation of neurofibrillary tangles, which are significant indicators of cellular disruption and cognitive decline [7]. Notably, this phenomenon has predominantly been reported in young opioid users [8]. This review aims to provide a comprehensive overview of how various substances impact Tau protein function, ultimately contributing to cognitive and neurological deficits.

Growing evidence indicates that Tau pathology in the context of SUDs arises from different but often converging molecular mechanisms. These include: (1) increased Tau phosphorylation due to overactivation of kinases such as GSK-3β, CDK5, and MAPKs; (2) decreased Tau dephosphorylation through the inhibition of phosphatases like PP1 and PP2A; (3) Tau cleavage mediated by activated caspase-3; and (4) altered Tau expression or trafficking influenced by neuroinflammation, oxidative stress, and disruptions in insulin signaling [9,10,11,12,13]. These changes frequently occur in brain regions implicated in executive function, memory, and reward processing—particularly the prefrontal cortex, hippocampus, and striatum—highlighting the relevance of regional vulnerability in Tau-mediated neurotoxicity.

These effects are substance-specific. For instance, alcohol and opioids induce Tau hyperphosphorylation and truncation, while psychostimulants such as cocaine and methamphetamine affect Tau levels through endosomal dysfunction and kinase overactivation. Cannabinoids and certain psychedelics, such as harmine or psilocybin, have demonstrated neuroprotective potential by reducing Tau phosphorylation or aggregation through receptor signaling and DYRK1A inhibition [10,14,15]. Depending on the substance, Tau can either accumulate in a toxic hyperphosphorylated form or be diminished in its normal function due to molecular destabilization, ultimately impacting cognitive function.

Cognitive function encompasses multiple domains, including memory, attention, executive functioning, processing speed, visuospatial abilities, and language [16]. Chronic exposure to psychoactive substances has been associated with domain-specific impairments. For example, alcohol and opioids are frequently linked to deficits in memory, visuospatial skills, and executive function [17,18,19,20]. Stimulants such as cocaine and methamphetamine often impair attention and cognitive flexibility [21,22], while dissociatives and psychedelics may disrupt working memory, emotional regulation, and psychomotor speed [23,24]. These domain-specific patterns may reflect differential Tau-related vulnerability across brain circuits and should guide future assessments and interpretations.

## 2. Results

**Figure 1:**
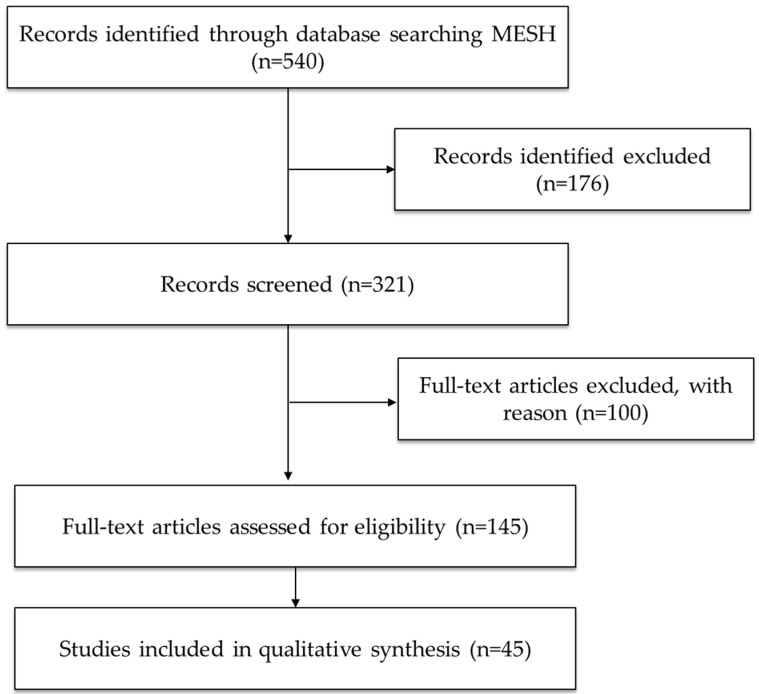
PRISMA flow diagram of the search and selection process.

Five hundred forty records were identified across the databases used, and 45 articles were selected for inclusion in this review based on the selection criteria described. Figure 1 shows the search and selection process of the scientific works included in this review.

Table 1 summarizes the characteristics and main findings from the selected studies, with a focus on Tau protein levels and cognitive outcomes.

### 2.1. Tau and Depressants (Alcohol)

Chronic and excessive alcohol consumption is strongly associated with cognitive decline and an elevated risk of dementia, often manifesting as deficits in visuospatial processing, memory, and executive function. While cognitive improvement may occur following sustained abstinence, the clinical distinction between alcohol-related cognitive impairment and early AD remains difficult in older adults, due to overlapping symptomatology and shared risk factors such as age, metabolic dysfunction, and neuroinflammation [46].

Neuropathological studies have identified neurofibrillary tangles—composed of hyperphosphorylated Tau—in the nucleus basalis of thiamine-deficient individuals with chronic alcoholism. However, such findings are absent in other brain regions or non-alcoholic controls [25]. In neurons with Tau tangles, a reduction in markers of microtubule stability, including acetylated α-tubulin and tubulin mRNA expression, has been observed [47], pointing to structural cytoskeletal disruptions that may serve as potential molecular biomarkers of alcohol-induced neurodegeneration.

Klotho, a transmembrane protein with antioxidants and anti-inflammatory properties, has emerged as a key neuroprotective factor. Lower circulating levels of α-Klotho have been documented in individuals with daily alcohol consumption, mirroring reductions reported in patients with alcohol-related brain atrophy [48,49]. In murine models, Klotho deficiency results in cognitive dysfunction and synaptic loss [50]. Moreover, an inverse relationship between Klotho levels and Tau pathology has been reported in AD patients [51], suggesting that chronic alcohol exposure may weaken endogenous neuroprotection and facilitate Tau accumulation.

Preclinical studies demonstrate that even nondependent alcohol consumption can exacerbate AD-like pathology. In C57BL/6J mice, voluntary alcohol intake altered the neuroproteome in the prefrontal cortex and amygdala, highlighting key AD-related proteins such as Tau (MAPT), APP, and PSEN-1, along with pathways involved in metabolism, cytoskeletal regulation, and oxidative stress. In 3xTg-AD mice, chronic alcohol exposure worsened cognitive deficits (e.g., impaired spatial memory, sensorimotor gating) and increased Aβ42/40 ratios and total Tau levels in entorhinal, prefrontal, and limbic regions. Notably, alcohol also reduced Akt/mTOR pathway signaling. These findings suggest that alcohol potentiates Tau pathology and accelerates AD-like neurodegeneration via disruption of neuroplasticity and stress-response mechanisms [28]. 

On the other hand, CSF biomarker analyses show that both AD and alcohol-related cognitive disorder (ARCD) are associated with elevated total Tau levels. Nonetheless, ARCD patients typically present with comparatively lower Tau concentrations, higher Aβ42 levels, and a more favorable Tau/Aβ42 ratio than those with AD [48]. These biochemical differences may help differentiate between the two conditions in aging populations, where heavy alcohol use constitutes a significant modifiable risk factor for dementia [52,53].

Experimental data also indicate that while Tau phosphorylation declines over time under normal conditions, ethanol exposure maintains elevated p-Tau levels, suggesting a persistent effect that may contribute to the cognitive alterations observed in fetal alcohol spectrum disorders (FASDs) [26].

Mechanistically, alcohol induces Tau pathology through the activation of GSK-3β and caspase-3-mediated Tau cleavage, generating aggregation-prone fragments. These alterations are observed across multiple brain regions, including the hippocampus, prefrontal cortex, and striatum, and are associated with microglial activation and neuroinflammatory cascades. Pharmacological inhibition of these pathways—e.g., with lithium—has been shown to attenuate these effects in preclinical models [54,55,56]. 

Beyond these kinase-dependent mechanisms, emerging evidence highlights the NLRP3 inflammasome as a critical mediator in Tau pathology. Hyperphosphorylated Tau not only accumulates into extracellular neurofibrillary tangles but can also activate the NLRP3 inflammasome within microglia, thereby amplifying neuroinflammatory responses [57]. Experimental studies in Tau transgenic mice have demonstrated that genetic deletion or pharmacological inhibition of *NLRP3* or its adaptor protein ASC results in reduced Tau phosphorylation and mitigated cognitive decline [58]. Furthermore, Tau22/Nlrp3–/– mice exhibit altered activity of Tau kinases and phosphatases, suggesting that the inflammasome may influence upstream regulatory pathways of Tau metabolism [59]. These findings position NLRP3 not merely as a downstream effector but as an active participant in the modulation of Tau dynamics.

Neuroinflammatory responses mediated by Toll-like receptor 4 (TLR4) and the NLRP3 inflammasome further potentiate Tau pathology through microglial activation and the release of IL-1β, IL-6, and TNF-α [60]. Notably, adolescent binge-like ethanol exposure in 3xTg-AD mice leads to sustained hippocampal p-Tau181 accumulation and neuroinflammation in adulthood, effects that are mitigated by anti-inflammatory agents such as minocycline [61].

Circuit-level investigations reveal that alcohol disrupts cortico-subcortical communication, particularly between the orbitofrontal cortex (OFC), medial prefrontal cortex (mPFC), and dorsal striatum. These alterations compromise cognitive flexibility, decision-making, and inhibitory control—key functions for adaptive behavior. Disruption of these pathways shifts behavioral control from goal-directed to habitual patterns, promoting compulsive alcohol-seeking and cognitive deterioration [62,63]. Additional regions, such as the hippocampus, anterior cingulate cortex (ACC), and dopaminergic midbrain nuclei, also show altered activity and synaptic plasticity, especially following adolescent exposure [64,65]. Such circuit remodeling likely underpins the persistent cognitive deficits observed despite abstinence. 

**Figure 2:**
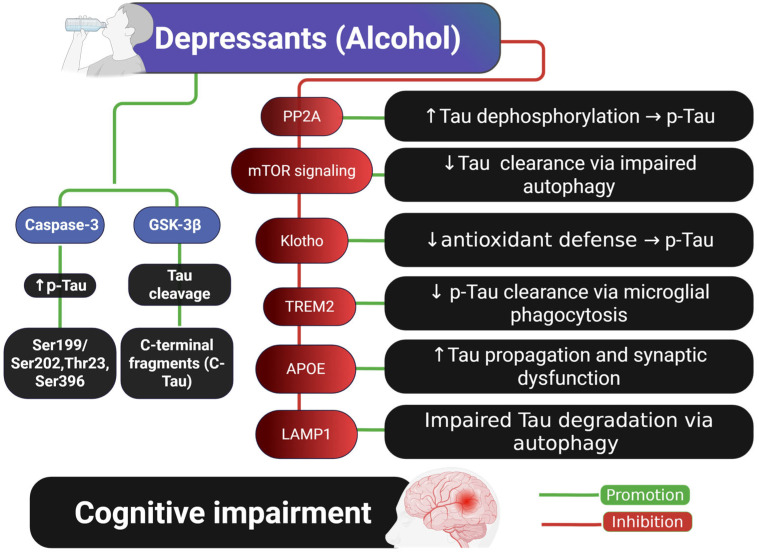
Schematic representation of the molecular mechanisms by which alcohol (a depressant) contributes to Tau pathology and cognitive impairment. Alcohol promotes Tau phosphorylation (p-Tau) via activation of Caspase-3 and GSK-3β, leading to Tau cleavage and the formation of C-terminal Tau fragments. Additionally, alcohol inhibits several key cellular pathways—PP2A, Klotho, TREM2, and LAMP1—which are typically involved in Tau dephosphorylation, antioxidant defense, microglial clearance, and autophagy, respectively. Dysregulation of these pathways contributes to Tau accumulation, aggregation, and impaired degradation, ultimately leading to cognitive dysfunction. Abbreviations and symbols: p-Tau: phosphorylated Tau; C-Tau: C-terminal Tau fragments; GSK-3β: glycogen synthase kinase-3 beta; PP2A: protein phosphatase 2A; mTOR: mechanistic target of rapamycin; TREM2: triggering receptor expressed on myeloid cells 2; APOE: apolipoprotein E; LAMP1: lysosomal-associated membrane protein 1; Klotho: longevity-associated protein; ↑: increase; ↓: decrease; →: leads to. Figure created with BioRender.com.

Taken together, these findings support a model in which chronic alcohol consumption disrupts multiple protective and regulatory pathways involved in Tau homeostasis, ultimately leading to progressive Tau accumulation, impaired clearance, and neurodegeneration, as illustrated in Figure 2.

However, important limitations remain. Much of the mechanistic evidence stems from rodent models that differ in genetic background, sex, and developmental timing of exposure. Few studies use contingent paradigms or self-administration models, limiting their translational relevance. Additionally, nutritional deficits (e.g., thiamine deficiency) in alcoholic individuals may confound interpretations of Tau-related outcomes [66].

Despite these constraints, the alcohol–Tau axis represents a promising translational target. Therapeutic strategies such as GSK-3β or NLRP3 inhibition, activation of phosphatases (e.g., PP2A), or modulation of Klotho signaling have shown preliminary efficacy in reversing Tau pathology. Future research should implement longitudinal designs that simulate binge or intermittent exposure patterns and integrate multimodal analysis of Tau species across functionally distinct brain regions. Behavioral phenotyping linked to cognitive domains, and the inclusion of sex, age, and nutritional variables, will be crucial to refine the clinical applicability of these findings.

### 2.2. Tau and Stimulants (Cocaine and Methamphetamine)

Similar to alcohol, stimulant drugs such as cocaine and methamphetamine have been associated with significant disruption of Tau homeostasis, exacerbating cognitive decline through multiple converging molecular mechanisms. In vivo studies have demonstrated that cocaine can induce Alzheimer-like hyperphosphorylation of Tau protein in the rat brain, potentially leading to cognitive dysfunction. This effect has been linked to increased activity of kinases such as GSK-3β and CDK5, as well as oxidative stress and neuroinflammatory responses, although these pathways remain underexplored in cocaine models [33]. Collectively, these findings suggest a plausible link between chronic cocaine use, Tau dysregulation, and impaired cognition.

In parallel, methamphetamine exposure has shown even more robust evidence of Tau pathology across various experimental platforms. Studies have demonstrated that methamphetamine increases the expression of α-synuclein, total Tau, and phosphorylated Tau (p-Tau) in SH-SY5Y cells, primary cultured neurons, and in vivo models. Notably, Tau knockout effectively inhibited methamphetamine-induced α-syn overexpression in mouse brains. Furthermore, eliminating either α-syn or Tau reduced methamphetamine-induced neurotoxicity, highlighting a pathological crosstalk between both proteins [67]. This Tau–α-synuclein interaction represents a converging pathway with implications for synaptic toxicity and protein aggregation disorders [68].

In addition to protein aggregation, methamphetamine alters intracellular signaling cascades implicated in Tau pathology. Xu et al. reported that methamphetamine exposure disrupts insulin signaling, leading to brain insulin resistance, as evidenced by the downregulation of insulin receptor substrate-1 and AKT serine 473, along with activation of GSK-3β [35]. Given that GSK-3β is a critical kinase for Tau phosphorylation and a central node in the insulin/PI3K/AKT pathway, its dysregulation promotes aberrant Tau modifications. This link is further supported by the therapeutic reversal of Tau phosphorylation using rosiglitazone (an insulin-sensitizing drug) and TWS119 (a GSK-3β inhibitor) [35,69]. Thus, metabolic dysfunction may synergize with kinase dysregulation to drive Tau-related neurotoxicity in methamphetamine users.

Moreover, methamphetamine has been shown to activate endoplasmic reticulum (ER) stress pathways that contribute to neuronal apoptosis. Specifically, it elevates levels of phosphorylated PERK and caspase-12, upstream regulators of Tau, and suggests that abnormal Tau phosphorylation via CDK5 plays a key role in this process [70]. CDK5, when aberrantly activated, not only phosphorylates Tau at pathological epitopes but also promotes apoptotic cascades that impair neuronal viability and cognitive function [71]. 

Taken together, these findings support a multifactorial model in which stimulant-induced Tau pathology arises from the convergence of kinase dysregulation (GSK-3β, CDK5), insulin resistance, protein aggregation, and ER stress. However, the translational relevance of these findings is constrained by the reliance on acute or subchronic exposure models in young male rodents or immortalized neuronal cultures, which do not adequately capture the chronic, compulsive, and binge-like consumption patterns observed in human stimulant users. Differences in dosage, administration routes, and exposure duration—along with the frequent exclusion of key variables such as sex, age, nutritional status, and polydrug use—further reduce generalizability. 

At the neural circuit level, repeated stimulant exposure has been shown to disrupt corticostriatal and hippocampal–prefrontal connectivity, impairing networks essential for executive function, behavioral flexibility, and goal-directed behavior. These alterations may underlie persistent cognitive deficits observed in clinical populations, including impairments in working memory, inhibitory control, and reversal learning. Region-specific analysis of Tau phosphorylation in these vulnerable circuits may provide critical insights into the relationship between network-level dysfunction and molecular pathology [72,73,74]. However, most preclinical studies investigating methamphetamine-induced Tau pathology rely on acute or subchronic exposure protocols, frequently using intraperitoneal administration of 2–10 mg/kg for 3 to 14 days in male C57BL/6J. These models, while mechanistically informative, fail to capture key features of chronic human use such as compulsive intake, relapse cycles, and sex-specific vulnerability. 

Despite these limitations, stimulant-based models offer a mechanistic framework to interrogate Tau-related pathways that may be therapeutically tractable. For example, targeting kinase activity (e.g., CDK5, GSK-3β), restoring insulin signaling, or modulating ER stress responses may represent promising strategies to attenuate Tau pathology. Future studies should prioritize contingent self-administration protocols, longitudinal behavioral assessments, and tauopathy-resilient vs. susceptible animal strains, coupled with region-specific quantification of Tau isoforms and aggregates. Additionally, sex-stratified and developmentally timed exposure models would clarify vulnerability windows and improve clinical translation.

**Figure 3:**
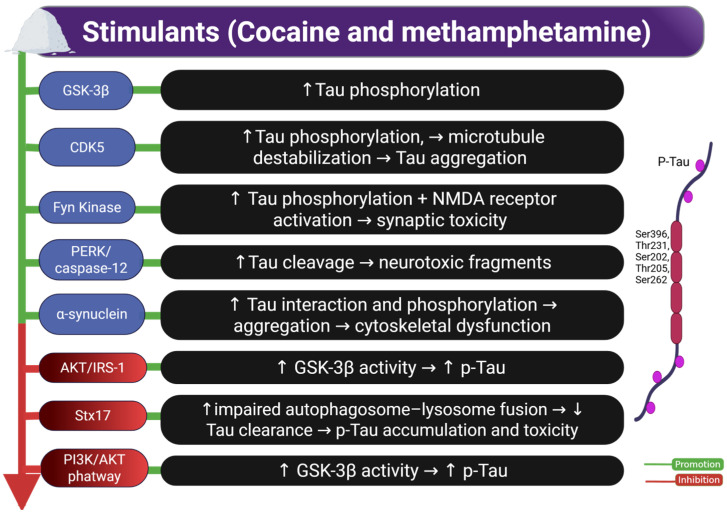
Effects of stimulants (cocaine and methamphetamine) on Tau phosphorylation and related molecular pathways. The image illustrates how exposure to stimulants such as cocaine and methamphetamine promotes Tau phosphorylation and neurotoxicity through various molecular mechanisms. Key pathways include GSK-3β and CDK5 activation, Fyn kinase-mediated NMDA receptor activation, and impaired autophagy via Stx17. Inhibition of insulin signaling pathways (AKT/IRS-1 and PI3K/AKT) further contributes to GSK-3β hyperactivation and p-Tau accumulation. These events lead to Tau cleavage, aggregation, cytoskeletal dysfunction, and synaptic toxicity, ultimately associated with cognitive impairment. Abbreviations and symbols: GSK-3β: glycogen synthase kinase-3 beta; CDK5: cyclin-dependent kinase 5; Fyn: non-receptor Src family tyrosine kinase; NMDA: N-methyl-D-aspartate; PERK: protein kinase RNA-like endoplasmic reticulum kinase; caspase-12: cysteine-aspartic protease 12; α-synuclein: alpha-synuclein; IRS-1: insulin receptor substrate-1; AKT: protein kinase B; Stx17: syntaxin 17; PI3K: phosphoinositide 3-kinase; p-Tau: phosphorylated Tau; ↑: increase; ↓: decrease; →: leads to. Figure created with BioRender.com.

In summary, although methodological limitations persist, stimulant-based research has delineated key molecular pathways susceptible to therapeutic intervention. Figure 3 provides an integrative overview of the molecular cascades implicated in Tau hyperphosphorylation and neurotoxicity induced by cocaine and methamphetamine. Targeting common molecular nodes, such as GSK-3β and CDK5, may offer promising avenues for therapeutic intervention. Nevertheless, future studies should prioritize the use of translationally relevant models and clinical cohorts to define better the pathological relevance of these mechanisms in human stimulant users.

### 2.3. Tau and Cannabinoids

The investigation into how cannabinoids influence Tau pathology and cognitive outcomes remains limited but increasingly relevant. Cannabis has been historically used for both medicinal and recreational purposes, with the two primary active cannabinoids being cannabidiol (CBD) and tetrahydrocannabinol (THC). Emerging evidence suggests that activation of cannabinoid receptors CB1 and CB2, through either natural or synthetic agonists, may exert neuroprotective effects by reducing Tau phosphorylation and enhancing intrinsic repair mechanisms in the brain [75]. 

This notion is supported by Aso et al., who demonstrated that chronic treatment with arachidonyl-2-chloroethylamide (ACEA), a CB1-selective agonist, significantly reduced p-Tau levels in mice [40]. These effects were most evident in the hippocampus and prefrontal cortex—regions critically involved in early tauopathies and AD, indicating a region-specific benefit [75].

Similarly, JWH133, a potent CB2 receptor agonist, was shown to reduce both Tau phosphorylation and GSK3β activity in HEK293 Tau-expressing cells. However, these effects were abolished when adenosine monophosphate-activated protein kinase (AMPK) activity was inhibited, implicating the AMPK/GSK3β pathway as a key regulatory axis [38]. This finding underscores the role of AMPK as an upstream modulator of Tau-related kinase activity in cannabinoid signaling.

Additionally, synthetic cannabinoids have been shown to attenuate glial-mediated inflammation and oxidative stress, which indirectly impact Tau phosphorylation. For instance, WIN55,212-2, a synthetic cannabinoid, inhibited Tau hyperphosphorylation in Aβ-stimulated PC12 cells, while also downregulating inducible nitric oxide synthase (iNOS) and nitric oxide production in C6 cells, suggesting anti-inflammatory and antioxidant effects that stabilize Tau homeostasis [76]. 

Beyond receptor-mediated signaling, CBD has been reported to interfere with Tau fibrillization and aggregation. It exerts this effect through multiple pathways, including mitochondrial protection, reduction of reactive oxygen species (ROS), and suppression of microglial activation—all processes tightly linked to neurodegenerative progression. Preclinical models have further supported CBD’s potential to attenuate Tau pathology and cognitive decline by modulating these mechanisms [37].

Despite these promising outcomes, most studies are based on in vitro systems or rodent models, often using acute or subchronic treatment durations under controlled conditions. These do not fully recapitulate the complex pharmacodynamics, receptor distribution, or long-term effects of cannabinoid use in humans. Importantly, CB1 receptors are predominantly expressed in the hippocampus, cerebellum, and frontal cortex, while CB2 receptors are primarily localized to microglia in pathological contexts. This regional specificity may influence the efficacy and safety profile of cannabinoid-based interventions [77].

Moreover, most experimental paradigms utilize synthetic cannabinoids with distinct pharmacokinetic and behavioral profiles compared to phytocannabinoids, such as THC and CBD used clinically or recreationally. This discrepancy poses a challenge in translating preclinical findings to human populations.

**Figure 4:**
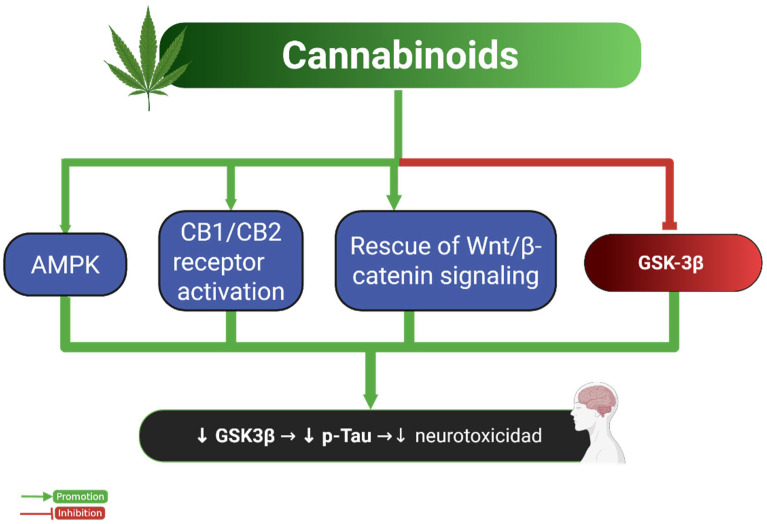
Neuroprotective effects of cannabinoids on Tau pathology. This diagram illustrates the molecular pathways through which cannabinoids reduce Tau phosphorylation and neurotoxicity. Cannabinoids activate AMPK and CB1/CB2 receptors, which in turn enhance Wnt/β-catenin signaling and inhibit GSK-3β activity. These interactions result in decreased p-Tau levels and reduced neurotoxicity. The green arrows indicate promotion, while the red lines indicate inhibition. Abbreviations and symbols: AMPK: AMP-activated protein kinase; CB1/CB2: cannabinoid receptor type 1 and type 2; Wnt: Wingless-related integration site; β-catenin: beta-catenin; GSK-3β: glycogen synthase kinase-3 beta; p-Tau: phosphorylated Tau; ↓: decrease; →: leads to. Figure created with BioRender.com.

Figure 4 integrates these findings into a conceptual framework, highlighting cannabinoid-mediated modulation of AMPK, GSK-3β, and Wnt/β-catenin pathways as potential therapeutic targets. Nevertheless, the psychotropic effects associated with CB1 agonists—especially in high doses—represent a major translational barrier, particularly in vulnerable populations [78,79]. Therefore, further rigorous studies are warranted to define optimal cannabinoid formulations, dosing strategies, and long-term safety profiles.

Although cannabinoid models suggest promising neuroprotective effects on Tau pathology, current evidence is constrained by methodological limitations. Most findings stem from acute exposure models or genetically modified rodents, which may not accurately reflect the pharmacokinetics and receptor selectivity of clinically relevant phytocannabinoids. Furthermore, strain-specific differences in CB1/CB2 receptor distribution and responsiveness may significantly affect outcomes. Future research should incorporate chronic exposure paradigms that mirror real-world therapeutic or recreational use, with a focus on long-term impacts on Tau phosphorylation, aggregation, and regional brain vulnerability—particularly in the hippocampus, prefrontal cortex, and entorhinal cortex.

### 2.4. Tau and Opioids

The accumulation of p-Tau has been consistently associated with cognitive dysfunction in individuals with opioid dependence, particularly morphine users. This pathological form of Tau contributes to hippocampal atrophy, oxidative stress, and apoptotic neuronal loss, representing a key neuropathological hallmark in opioid-related cognitive impairments [80].

Opioid-induced dysregulation of the endogenous opioid system—comprising opioid receptors and peptides—may exacerbate neuroinflammation and Tau hyperphosphorylation, accelerating the degeneration of cholinergic neurons essential for memory and executive function [81].

Postmortem studies of opioid users have reported increased p-Tau deposition in brain regions commonly affected during early AD, including the frontal and temporal cortices and the locus coeruleus. These alterations correlate with activated microglia and pro-inflammatory states, suggesting that opioid use may initiate or amplify early neurodegenerative processes [42,82]. Neuroimaging data further support this link, revealing cortical thinning, hippocampal volume loss, and white matter disruption in chronic opioid users, paralleling Tau-associated neurodegeneration and related cognitive deficits [83,84].

Taken together, these findings suggest that opioids promote Tau pathology through a multifactorial mechanism involving the activation of kinases such as GSK-3β [42], CDK5 [13], and JNK/p38 MAPK [9]; the inhibition of phosphatases like PP1 via KEPI upregulation [12]; microglial activation and neuroinflammation [11,42]; and disruption of opioid receptor signaling, which affects neuronal survival and plasticity [81]. Specifically, chronic opioid exposure leads to μ-opioid receptor downregulation, disrupted opioid peptide homeostasis, and diminished neurotrophic support via the PI3K/Akt pathway, contributing to BDNF reduction and disinhibition of GSK-3β, which further enhances Tau phosphorylation [78,85]. 

Collectively, these mechanisms converge on Tau hyperphosphorylation, synaptic dysfunction, and progressive neurodegeneration—mimicking AD-like pathology in the context of substance use.

Despite the strong biological plausibility, existing evidence is largely based on rodent and in vitro models that often fail to reflect the complexity of chronic opioid dependence in humans—characterized by prolonged use, polydrug combinations, and psychiatric comorbidities. Postmortem human studies are also limited by small sample sizes, confounding variables, and lack of prospective clinical data, limiting their generalizability and causal inference.

**Figure 5:**
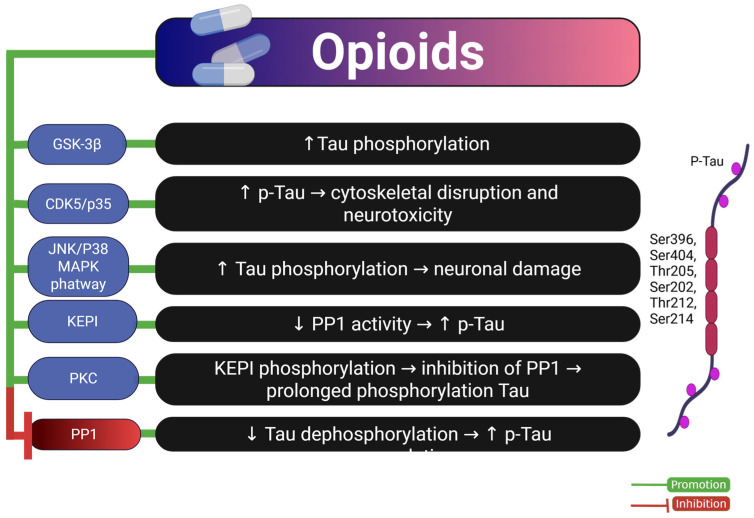
Mechanisms of opioid-induced Tau pathology. The diagram summarizes the molecular pathways by which opioids promote Tau hyperphosphorylation and neurotoxicity. Opioid exposure enhances the activity of kinases such as GSK-3β, CDK5/p35, and JNK/p38 MAPK, leading to increased Tau phosphorylation. Additionally, PKC-induced phosphorylation of KEPI inhibits PP1, a phosphatase responsible for Tau dephosphorylation, resulting in p-Tau accumulation. Overall, this contributes to cytoskeletal disruption and neuronal damage. Green arrows indicate promotion; red lines indicate inhibition. Abbreviations and symbols: GSK-3β: glycogen synthase kinase-3 beta; CDK5/p35: cyclin-dependent kinase 5 with its activator p35; JNK/p38 MAPK: c-Jun N-terminal kinase/p38 mitogen-activated protein kinase; KEPI: kinase enhanced phosphatase inhibitor; PKC: protein kinase C; PP1: protein phosphatase 1; p-Tau: phosphorylated Tau; ↑: increase; ↓: decrease; →: leads to. Figure created with BioRender.com.

Figure 5 summarizes the complex molecular mechanisms by which opioids influence Tau pathology, identifying kinase activation, phosphatase inhibition, and glial-mediated inflammation as key therapeutic targets. However, to increase translational relevance, future research should focus on longitudinal human cohorts and comparative studies using chronic exposure protocols to distinguish opioid-specific Tau alterations from those induced by other substances.

Most opioid-related preclinical studies rely on acute morphine administration under simplified laboratory conditions, typically involving single injections (5–10 mg/kg, s.c.) or short-term regimens (3–7 days) in rodents such as C57BL/6J or Sprague-Dawley rats. However, these protocols fall short of replicating the chronic, escalating, and polydrug consumption patterns observed in human opioid use disorder. In real-world contexts, opioid use often entails prolonged exposure, co-use with other central nervous system depressants (e.g., benzodiazepines), and comorbid infections such as HIV, all of which can interact with and modulate Tau pathology. To address this complexity, some models have incorporated dual-exposure paradigms—such as chronic morphine administration in combination with HIV-1 Tat protein infusion in transgenic mice—which synergistically exacerbate Tau phosphorylation, neuroinflammation, and cognitive decline. These approaches offer a more ecologically valid framework for understanding opioid-related neurodegeneration.

Although these limitations persist, opioid-based models offer valuable insights into kinase–phosphatase imbalance, inflammatory mechanisms, and neurotrophic disruption contributing to Tau pathology. Future studies should expand to include prescription opioids (e.g., oxycodone, fentanyl) to assess differential effects on Tau isoforms, truncation, and aggregation profiles.

Moreover, multimodal approaches—combining neuroimaging (e.g., Tau PET), cerebrospinal fluid biomarkers, and transcriptomic/phosphoproteomic profiling—will be essential to delineate opioid-specific contributions to Tauopathy and guide targeted interventions in opioid-related cognitive decline.

### 2.5. Tau and Dissociative Drugs

Ketamine, an NMDA (N-methyl-D-aspartate) receptor antagonist, exhibits rapid antidepressant effects at subanesthetic doses by blocking glutamatergic signaling, leading to a transient altered mental state [86]. Despite its therapeutic potential, chronic ketamine use has been associated with neurotoxicity, including increased p-Tau accumulation, synaptic dysfunction, and cognitive impairments in both rodent and non-human primate models [45,87]. Ketamine’s pharmacological profile involves modulation of multiple receptor systems beyond NMDA, including the α-amino-3-hydroxy-5-methyl-4-isoxazole-propionic acid (AMPA) receptors, which undergo compensatory hyperactivation following NMDA receptor blockade [44,88,89]. This receptor shift leads to dysregulated calcium influx and activation of Tau-related kinases such as GSK-3β and CDK5, which drive pathological phosphorylation at sites including Ser202/Thr205 and Ser396, commonly implicated in tauopathies [44,90,91].

Recent studies suggest that ketamine-induced Tau phosphorylation is not only site-specific but also functionally linked to downstream synaptic disruption [92]. For instance, long-term ketamine exposure in wild-type and Tau knockout mice resulted in selective hyperphosphorylation at Ser202/Thr205 and Ser396, but not at other residues such as Ser199, Ser262, or Ser404. Moreover, a Tau-dependent reduction in AMPA receptor expression was observed in the hippocampal membrane, suggesting that phosphorylated Tau negatively modulates synaptic receptor localization and function [44].

These effects predominantly affect the hippocampus and prefrontal cortex, brain regions critical for learning, memory, and executive function. Dendritic spine loss and decreased synaptic density have been observed in these areas following prolonged ketamine exposure, which may explain the persistent cognitive deficits seen in both preclinical models and clinical case reports [93,94,95,96].

Beyond receptor signaling, ketamine appears to impair endosomal-lysosomal function, a key mechanism for Tau degradation. Studies show that ketamine induces delirium-like behaviors in mice, elevates serum Tau levels, and leads to the accumulation of immature endosomes marked by increased Rab5 and Rab7, while simultaneously inhibiting their maturation [97]. This results in the cytoplasmic retention of Tau within endosomes and increased Tau release, suggesting a possible route for trans-synaptic propagation of pathology.

Disruption of the endosomal-lysosomal pathway is increasingly recognized as a hallmark of neurodegenerative diseases. Ketamine-induced impairment of Rab GTPase activity may therefore exacerbate Tau aggregation and spread, particularly under conditions of repeated or high-dose exposure [91,97,98].

**Figure 6:**
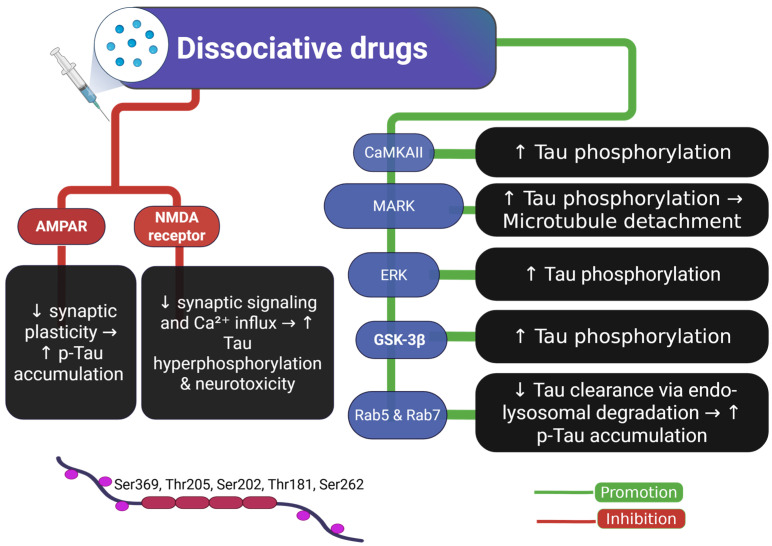
Dissociative drugs and their effects on Tau phosphorylation and neurotoxicity pathways. This figure illustrates the mechanisms through which dissociative drugs modulate Tau pathology. Inhibitory effects on AMPAR and NMDA receptors lead to decreased synaptic plasticity and calcium influx, promoting Tau hyperphosphorylation and neurotoxicity. Simultaneously, activation of downstream kinases such as CaMKII, MARK, ERK, and GSK-3β promotes Tau phosphorylation, while impaired Rab5/Rab7-mediated endo-lysosomal degradation contributes to p-Tau accumulation. Green arrows indicate promotion, and red lines indicate inhibition of the indicated process. Abbreviations and symbols: AMPAR: α-amino-3-hydroxy-5-methyl-4-isoxazolepropionic acid receptor; NMDA: N-methyl-D-aspartate receptor; CaMKII: calcium/calmodulin-dependent protein kinase II; MARK: microtubule affinity-regulating kinase; ERK: extracellular signal-regulated kinase; GSK-3β: glycogen synthase kinase-3 beta; Rab5/Rab7: Ras-related proteins involved in endosomal trafficking; p-Tau: phosphorylated Tau; Ca^2+^: calcium ion; ↑: increase; ↓: decrease; →: leads to. Figure created with BioRender.com.

Figure 6 integrates these findings by illustrating how dissociative drug exposure disrupts synaptic receptor balance, promotes Tau phosphorylation, and impairs intracellular degradation pathways—cumulatively contributing to neurodegeneration.

Despite growing mechanistic evidence, most studies rely on preclinical models that lack translational validation. Dose, frequency, developmental timing, and comorbid substance use vary greatly between experimental paradigms and clinical settings, complicating extrapolation. Some protocols employ chronic or supratherapeutic doses not representative of human antidepressant use, limiting external validity.

To improve translational relevance, future studies should replicate realistic ketamine exposure paradigms, including intermittent dosing schedules, long-term use, and combined exposure with other drugs or stressors. Moreover, time-resolved analyses of Tau phosphorylation, isoform distribution, and aggregation in key regions like the hippocampus, entorhinal cortex, and medial prefrontal cortex are essential. This should be coupled with behavioral assessments relevant to cognitive domains affected by Tau dysregulation, such as working memory, executive function, and affect regulation.

Advanced models should incorporate sex and age as biological variables, track autophagic flux and Rab-mediated endosome dynamics, and explore reversibility of Tau pathology post-withdrawal.

Human-relevant approaches—including iPSC-derived neurons, organoids, and Tau PET imaging—may help clarify whether Tau alterations reflect a causal driver or a downstream response to ketamine-induced neurotoxicity. Ultimately, such integrative strategies are needed to determine whether modulating Tau-related pathways can mitigate long-term cognitive risks associated with dissociative drug exposure.

### 2.6. Tau and Psychedelic Drugs

Research on the correlation between Tau protein and psychedelic drugs remains limited. However, emerging findings suggest that compounds such as LSD, psilocybin, and dimethyltryptamine (DMT) may significantly influence Tau metabolism and neuronal function. 

In an in vitro study using SH-SY5Y cells—a human adrenergic neuroblastoma line—investigators tested the hypothesis that D-lysergic acid diethylamide (LSD) alters microtubule-associated Tau dynamics. Treatment with LSD at concentrations of 10^−5^ and 10^−7^ M for 48 hours led to a reduction in the 50 kDa Tau isoform in both cytoplasmic and membrane compartments [14]. This observation suggests that LSD may promote Tau detachment from microtubules, resulting in elevated levels of soluble Tau and potentially compromising microtubule integrity and axonal transport. Such destabilization may affect neuronal communication and viability, particularly in brain regions with high synaptic remodeling demands, such as the prefrontal cortex and hippocampus [99,100].

Beyond LSD, other serotonergic psychedelics, particularly psilocybin, appear to exert neuroprotective effects in experimental models. In a rodent model of repetitive mild traumatic brain injury (rmTBI), psilocybin administration reduced phosphorylated Tau (PHF-1 epitope) levels in the soluble fraction and showed a downward trend in insoluble aggregated Tau [15]. These biochemical changes correlated with improved cognitive and motor performance, likely mediated by reversal of neuroinflammation, restoration of cerebrovascular reactivity, and activation of the BDNF–TrkB signaling axis. Notably, psilocybin treatment enhanced dendritic spine density and increased BDNF expression in the hippocampus and sensorimotor cortex—regions critical for both cognitive flexibility and motor control [15,101]. 

Additionally, harmine and other β-carboline alkaloids—compounds structurally related to DMT—have shown the ability to reduce Tau phosphorylation at key residues such as S396, S262/S356, and T231 through inhibition of DYRK1A, a kinase implicated in AD pathology [10].

Harmine-mediated DYRK1A inhibition has also been shown to regulate neurogenesis and axonal growth, especially in the dentate gyrus of the hippocampus. Furthermore, DYRK1A suppression leads to downstream modulation of GSK-3β and MAPK/ERK pathways, indirectly reducing aberrant Tau phosphorylation [102,103].

**Figure 7:**
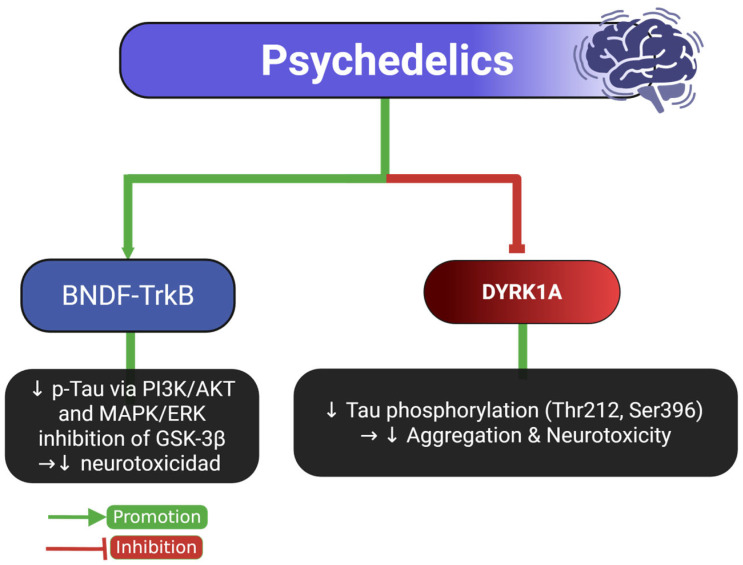
Psychedelics and their dual regulatory role in Tau phosphorylation pathways. This diagram illustrates how psychedelics influence Tau phosphorylation through both neuroprotective and neurotoxic pathways. Activation of BDNF-TrkB signaling reduces Tau phosphorylation by modulating PI3K/AKT and MAPK/ERK pathways, which inhibit GSK-3β, ultimately decreasing neurotoxicity. Conversely, psychedelics also inhibit DYRK1A, a kinase that typically promotes Tau phosphorylation at Thr212 and Ser396, leading to reduced aggregation and neurotoxicity. Green arrows indicate promotion, and red lines indicate inhibition. Abbreviations and symbols: BDNF: brain-derived neurotrophic factor; TrkB: tropomyosin receptor kinase B; PI3K/AKT: phosphoinositide 3-kinase/protein kinase B pathway; MAPK/ERK: mitogen-activated protein kinase/extracellular signal-regulated kinase; GSK-3β: glycogen synthase kinase-3 beta; DYRK1A: dual-specificity tyrosine-phosphorylation-regulated kinase 1A; p-Tau: phosphorylated Tau; ↓ decrease; →: leads to. Figure created with BioRender.com.

At the molecular level, psychedelics appear to exert their modulatory effects through serotonergic (5-HT2A) receptor activation, which triggers intracellular cascades involving PI3K/AKT, MAPK/ERK, and mTOR signaling. These pathways converge on Tau-related kinases (GSK-3β, DYRK1A, CDK5), promoting either its stabilization or clearance depending on the context. Additionally, psychedelics may enhance autophagy and mitochondrial bioenergetics, both of which are crucial for Tau homeostasis and synaptic maintenance [104,105]. These molecular pathways are summarized in Figure 7.

Despite the encouraging findings, most current evidence is derived from in vitro systems or short-term animal models that may not reflect the complexity of human psychedelic use. Variability in Tau epitopes analyzed, administration routes, and compound doses complicates cross-study comparisons, while the functional consequences of Tau alterations remain speculative without behavioral validation. To improve translational value, future research should implement chronic and intermittent exposure paradigms reflecting therapeutic and recreational patterns, include region-specific quantification of Tau isoforms and phosphorylation states, and incorporate functional assessments of synaptic plasticity and cognition. Comparative studies between classical serotonergic psychedelics (e.g., LSD, psilocybin) and β-carbolines (e.g., harmine) may help clarify their differential modulation of DYRK1A and related pathways. An integrated approach combining behavioral assays, neuroimaging, and molecular profiling will be essential to determine whether psychedelics represent viable tools for modifying Tau-associated neurodegenerative processes or trauma-related tauopathies.

## 3. General Considerations

The interplay between Tau pathology and psychoactive substance use represents a compelling yet complex neurobiological intersection. Across the studies reviewed, a recurring observation is the presence of Tau hyperphosphorylation following chronic exposure to substances such as alcohol, opioids, stimulants, dissociatives, and psychedelics. However, interpreting this convergence requires a nuanced understanding of both the temporal dynamics and anatomical specificity of Tau alterations within the brain’s cognitive circuitry.

One critical question is whether Tau dysfunction acts as an initiator of neurotoxicity or arises as a downstream effect of broader neuroadaptive changes. The temporal patterns differ among substances: in methamphetamine and opioid exposure, Tau abnormalities appear rapidly, suggesting a potential role in triggering early synaptic disruption. In contrast, alcohol and ketamine models frequently demonstrate persistent Tau accumulation long after cessation, indicating that Tau may amplify or sustain damage following initial circuit remodeling. These observations support a dualistic role for Tau—both as an early effector and as a chronic driver of neurodegeneration—depending on the neurochemical context and exposure duration.

This temporal ambiguity underscores the need for longitudinal studies incorporating self-administration paradigms and repeated neurobehavioral evaluations. The inclusion of region-specific Tau mapping across brain structures—particularly the hippocampus, prefrontal cortex, and striatum—will be essential for temporally linking Tau alterations with evolving cognitive phenotypes. Behavioral domains such as decision-making, working memory, and cognitive flexibility should be prioritized to delineate substance-specific profiles of impairment.

A synthesis of the available preclinical and clinical findings reveals that Tau pathology follows regionally distinct patterns depending on the substance involved. Alcohol and ketamine appear to primarily target the prefrontal cortex and hippocampus, both of which are critical for executive function and memory consolidation. In contrast, methamphetamine exposure is associated with Tau accumulation in the striatum and limbic areas, aligning with impairments in reward processing and affect regulation. Opioids tend to affect regions such as the locus coeruleus and temporal cortices, which are linked to arousal and emotional memory. These topographical differences may underlie the divergent neuropsychiatric outcomes seen across substance classes, offering a framework to interpret how Tau-driven disruptions translate into specific cognitive and affective profiles.

Although direct circuit-level studies remain scarce, the selective vulnerability of certain brain regions suggests that Tau accumulation may disrupt the bidirectional flow of information between cortical and subcortical structures. In particular, Tau-induced pathology in associative cortices—such as the prefrontal and temporal lobes—could impair top-down modulation of subcortical reward circuits, exacerbating impulsivity, craving, and maladaptive decision-making. This conceptual framework supports the hypothesis that Tau dysfunction not only reflects cellular toxicity but may actively compromise functional connectivity across large-scale networks relevant to addiction pathology.

Beyond timing, anatomical resolution is also critical. Although Tau hyperphosphorylation is observed across drug classes, the regional distribution of Tau pathology appears substance-specific. For example, alcohol and opioids primarily affect cortical regions associated with executive function and episodic memory, while stimulants preferentially disrupt subcortical areas related to reward processing and impulsivity. Dissociatives like ketamine target both cortical and subcortical regions but appear to exert a marked impact on entorhinal-hippocampal AMPAR signaling. Psychedelics, though less extensively studied, may exert region-specific effects via serotonergic and neurotrophic pathways in the prefrontal cortex and hippocampus. This topographic variation suggests that Tau-mediated dysfunction reflects distinct circuit vulnerabilities, with downstream consequences that align with the behavioral syndromes characteristic of each drug class.

However, the translational relevance of these findings remains limited by methodological heterogeneity. Most studies rely on simplified in vitro systems or acute in vivo exposures, which may not accurately reflect the chronic, polydrug, and comorbid profiles of real-world substance use disorders. Furthermore, the diversity in Tau species evaluated—ranging from total to phosphorylated or truncated forms—complicates cross-study comparisons. Many investigations also lack adequate controls for confounders such as age, sex, genetic background, and psychiatric comorbidities, and often omit negative results, introducing potential bias into the literature.

Another major limitation is the inconsistent assessment of cognitive outcomes. In numerous studies, behavioral impairments are inferred from molecular alterations without direct validation, and when present, cognitive testing is often restricted to narrow domains. A shift toward multidimensional behavioral characterization—paired with molecular profiling—would enable more robust correlations between Tau dynamics and functional deficits.

Importantly, there is a growing need to consider modulatory factors that influence Tau vulnerability. Sex-specific differences in Tau phosphorylation and neurodegeneration have been documented in other contexts, yet are seldom addressed in substance use models. Similarly, age at onset, cumulative exposure, and individual resilience factors such as cognitive reserve may modulate the impact of Tau pathology. Integrating these variables into future research could enhance the clinical applicability of findings and support the development of targeted interventions.

An emerging direction involves comparing Tau dysregulation in substance users with its behavior in normative aging. Studies have demonstrated that in healthy individuals, Tau interacts with adaptive processes such as cortical plasticity and synaptic remodeling, contributing to cognitive resilience. Contrasting these physiological roles with the pathological patterns observed in substance use disorders may provide valuable insight into the tipping point at which Tau transitions from a functional modulator to a driver of dysfunction.

Ultimately, the field would benefit from harmonizing methodologies across laboratories, including standardized protocols for substance exposure, Tau quantification, and neurocognitive testing. Establishing consensus on key biomarkers and experimental designs will facilitate meta-analytic synthesis and foster clearer conclusions regarding the role of Tau in addiction-related neurotoxicity.

Despite existing gaps, the convergence on Tau dysregulation across drug classes highlights its potential as a biomarker of cumulative neural stress. Whether this represents a modifiable target for therapeutic intervention remains uncertain, but the evidence justifies further exploration. Future studies should pursue multimodal approaches, combining neuroimaging, fluid biomarkers, and transcriptomic profiling, to establish the relevance of Tau pathology in the human substance use context and evaluate its utility as a predictive marker or treatment endpoint.

## 4. Materials and Methods

### 4.1. Search Strategy

We included studies involving human and animal models exposed to psychoactive substances classified as depressants, stimulants, opioids, cannabinoids, dissociatives, or psychedelics. These categories were chosen due to their established or emerging evidence of altering Tau protein dynamics.

A comprehensive search of MEDLINE, PubMed, and the Cochrane Library was conducted for English-language publications up to June 2025. Based on the Population, Exposure, Comparison, Outcome (PECO) research question method, studies were included if they: (1) involved general population samples or specific subpopulations with a history of substance abuse; (2) examined the impact of substance abuse on the levels of Tau; (3) compared this biomarker level and cognitive function before and after exposure to substance abuse; and (4) reported quantitative data on these outcomes. Studies were excluded if they involved non-representative samples (e.g., patient groups, specific ethnic groups, high-risk subpopulations) or did not report quantitative data on the impacts of substance abuse on the specified biomarker and cognitive function. The search terms included keywords related to “Tau protein” OR MAPT OR tauopathy AND “substance abuse” OR “drug use” OR “alcohol use” OR “drug addiction” OR “methamphetamine” OR “cocaine” OR “opioids” OR “cannabis” OR “psychoactive substances”.

### 4.2. Study Selection and Analysis

Two reviewers independently conducted title and abstract screening. When the relevance of neurotrophic factors or study design could not be determined from abstracts alone, full texts were reviewed. Discrepancies are resolved through discussion or consultation with a third reviewer. For each study, data extracted included study aims, design, setting, year of data collection, outcome measures, effect sizes, and critical results. Data extraction was performed by one reviewer and verified by a second. Due to the small number of studies and variability in outcomes, meta-analyses were not conducted; instead, findings were summarized narratively.

## 5. Conclusions

Tau hyperphosphorylation and aggregation are well-established hallmarks of neurodegenerative diseases, most notably AD. Emerging evidence now implicates Tau dysregulation as a downstream consequence—and potentially a mechanistic driver—of neurobiological alterations induced by chronic exposure to psychoactive substances. Across drug classes including alcohol, opioids, stimulants, cannabinoids, dissociatives, and psychedelics, converging findings reveal that repeated substance use perturbs Tau homeostasis through diverse molecular routes, including dysregulation of kinases and phosphatases, oxidative stress, neuroinflammation, mitochondrial dysfunction, impaired autophagy, and disrupted synaptic plasticity.

This review systematically categorized available studies by experimental model and drug type, delineating how these substances impact Tau phosphorylation and related cognitive functions. Alcohol, methamphetamine, and opioids consistently elicited Tau hyperphosphorylation in cortical and subcortical regions tied to executive function, reward processing, and memory. In contrast, certain cannabinoids and psychedelics demonstrated potential neuroprotective properties, modulating Tau-related signaling in ways that reduced aberrant phosphorylation and enhanced synaptic resilience in preclinical models. Specifically, compounds such as harmine and psilocybin have shown efficacy in attenuating Tau pathology through mechanisms including DYRK1A inhibition and BDNF-TrkB pathway activation.

Nonetheless, substantial heterogeneity across studies—in terms of substance type, dosing regimens, exposure duration, brain region analyzed, Tau epitopes measured, and behavioral endpoints—limits translational interpretation. Most available data derive from acute or subchronic models, which may not reflect the progressive nature of Tau pathology in substance use disorders (SUDs). Moreover, critical factors such as sex, age, genetic background, and polysubstance exposure remain insufficiently addressed.

Future research should prioritize the adoption of longitudinal and region-specific models that recapitulate chronic substance exposure, incorporating time-resolved assessment of Tau species alongside cognitive and synaptic measures. Underexplored signaling pathways such as AMPK and Wnt/β-catenin warrant mechanistic dissection, while emerging therapeutic targets—including CB2 receptor agonists and DYRK1A inhibitors—require validation in human populations. Parallel efforts to standardize protocols for Tau quantification and behavioral phenotyping will also enhance reproducibility and meta-analytic rigor.

Collectively, current findings position Tau as a critical nexus in the neurotoxic effects of psychoactive substances. Its altered phosphorylation state may serve not only as a biomarker of cumulative neural insult but also as a modifiable mediator of cognitive dysfunction. Targeting Tau-related pathways offers a compelling strategy for mitigating substance-induced neurodegeneration, with translational implications that extend beyond addiction to broader neuropsychiatric and neurodegenerative contexts.

## Figures and Tables

**Table 1:** Effects of substances on Tau protein and cognitive impairment.

Substance	Model	Effects on Tau	Cognitive Impact	Proposed Mechanism	References
Depressants (Alcohol)	Cell culture (Human neurons)	↑ p-Tau, ↑ Neurofibrillary tangles in nucleus basalis	Memory dysfunction	Disturbance in phosphorylation/dephosphorylation pathways	[25]
Depressants (Alcohol)	Cell culture (neonatal rat hippocampus)	↑ p-Thr231-Tau	N/A	GSK-β activation, PP2A inhibition	[26]
Depressants (Alcohol)	Animal (C57BL/6J, 3xTg-AD mice)	↑ Total Tau, ↑ p-Tau (Ser199/Ser202)	Spatial memory deficits, impaired gating	Dysregulation of mTOR, CRMP2, HSPs, AMPH, GSK	[27,28,29,30]
Depressants (Alcohol)	Cerebrospinal fluid (Humans)	↑ Tau/Aβ42 and p-Tau/Aβ42 ratios in individuals with high adherence to the Mediterranean-alcohol dietary pattern	Preclinical AD risk	Associated with Aβ positivity, Tau/Aβ imbalance	[31]
Stimulants (Cocaine)	Animal (rats, mice)	↑ p-Tau (PHF-1), ↓ Tau-1, ↑ total Tau	Neurocytoskeletal changes; cocaine-cue memory	Alternative kinases, Fyn kinase activation, PI3K-AKT suppression	[32,33]
Stimulants (Cocaine)	Human-derived neuronal cell culture	↓ Tau (50 kDa)	N/A	Cytoskeletal instability	[34]
Stimulants (Methamphetamine)	Human cells (SH-SY5Y human neuroblastoma)	↑ p-Tau (Ser396, Thr231), ↑ total Tau; ER stress markers (p-PERK, caspase-12); disrupted insulin signaling (↓ IRS-1, AKT, ↑ GSK3β); ↓ autophagy	Neuronal apoptosis, not directly	CDK5 activation; α-syn interaction; ER stress; insulin signaling disruption; autophagy impairment	[35,36]
Stimulants (Methamphetamine)	Animal cell (Primary rat hippocampal neurons; Neuro2A mouse neuronal cells)	↑ p-Tau (Ser396, Thr231), ↑ total Tau; disrupted autophagy	Neurodegeneration, not directly	Tau hyperphosphorylation via CDK5 and α-syn; autophagy dysfunction	[36]
Cannabinoids	Human (recombinant human Tau protein 1N/4R; aggregation assays in vitro	CBD inhibits Tau fibril formation, prevents conformational changes	Neuroprotective potential	Direct interaction with Tau; competes with heparin at VQIINK/VQIVYK motifs	[37]
Cannabinoids	Human cells (HEK293)	HEK293 Tau (human embryonic kidney cells)	HEK293 Tau (human embryonic kidney cells)	HEK293 Tau (human embryonic kidney cells)	[38]
Cannabinoids	Animal cells (PC12 cells + Aβ)	CBD ↓ Tau hyperphosphorylation	Neuroprotection (in vitro)	Rescue of Wnt/β-catenin signaling	[39]
Cannabinoids	Animal models	↓ p-Tau (Thr181) in AβPP/PS1 transgenic mice	Prevented cognitive impairment	CB1 activation → ↓ GSK3β → ↓ Tau p	[40]
		↓ Tau and Aβ deposition; ↑ autophagy in PK-/-/TauVLW mice (FTD, Parkinsonism)	↓ Abnormal behaviors	CB1/CB2 activation → ↓ oxidative stress, ↑ mitochondrial function and autophagy	[41]
Opioids	Human	↑ p-Tau (AT8, AT100), NFTs in frontal/temporal cortex and locus coeruleus, and ↑ GSK3β in postmortem human brains	Cognitive impairment; overlaps with early AD pathology	↑ GSK3β activity; neuroinflammation; microglial activation correlates with p-Tau levels	[42]
		↑ p-Tau in AD-related regions (e.g., hippocampus) of postmortem brain tissue from heroin users	Potential predisposition to accelerated AD-like neurodegeneration	Microglial activation; p-Tau independent of duration of drug use	[11]
		↑ KEPI expression → PP1 inhibition in morphine-treated human brain tissue	Not specified	μ-opioid receptor activation → ↑ PKC → ↑ KEPI → PP1 inhibition → ↑ Tau phosphorylation	[12]
Opioids	Mouse model (Tat-transgenic) with morphine co-exposure	↑ pSer396 (striatum, PFC); Tat ↑ pSer404 and pThr205	Cognitive/sensory dysfunction (suggested)	↑ CDK5/p35 activity; region-specific Tau hyperphosphorylation	[13]
Opioids	Cell (rat embryo cortical neurons treated with morphine)	↑ Tau hyperphosphorylation	Not assessed	Opioid receptor-dependent activation of JNK/p38 MAPK pathway	[9]
Dissociative drugs (ketamine)	Animal models	↑ p-Tau (Ser396, Ser262, Thr181, Ser202/Thr205) dose-dependent in mouse	N/A	CaMKII activation → ↑ p-Tau; also affects MARK, ERK, GSK3 pathways	[43]
		↑ p-Tau (Ser202/Thr205, Ser396) in mice	↓ AMPA receptor levels, ↓ synaptic efficiency	Tau-dependent reduction in AMPA receptors; Tau phosphorylation mediates synaptic dysfunction	[44]
		↑ p-Tau in prefrontal and entorhinal cortex; TUNEL+ neurons in mice and monkeys	Memory impairment (linked to aging/Alzheimer-like neurodegeneration)	NMDA receptor antagonism → Tau hyperphosphorylation and potential apoptosis	[45]
Psychedelics	Human cell (SH-SY5Y neuroblastoma cells)	↓ Microtubule-associated Tau in both cytoplasmic and membrane fractions	N/A	Promotes Tau dissociation from microtubules, increasing soluble Tau levels	[14]
Psychedelics	Animal model (Female Wistar rats	↓ Phosphorylated Tau (PHF-1 epitope) to control levels in soluble fraction; ↓ trend in insoluble aggregated Tau	Improved cognitive and motor behaviors post-injury	Psilocybin enhances neuroplasticity, reduces neuroinflammation, restores vascular reactivity, and modulates BDNF-TrkB signaling	[15]
Psychedelics	Cell culture and in vitro assays (harmine and β-carbolines)	↓ Tau phosphorylation at S396, S262/S356, and T231	N/A	Inhibition of DYRK1A kinase by harmine and related β-carboline compounds	[10]

Abbreviations and symbols in the table: ↑ = increase; ↓ = decrease; p-Tau = phosphorylated Tau; NFTs = neurofibrillary tangles; CDK5 = cyclin-dependent kinase 5; GSK3β = glycogen synthase kinase 3 beta; PP2A/PP1 = protein phosphatases 2A and 1; CRMP2 = collapsin response mediator protein 2; AMPK = AMP-activated protein kinase; DYRK1A = dual-specificity tyrosine-phosphorylation-regulated kinase 1A; ERK = extracellular signal-regulated kinase; MARK = microtubule affinity-regulating kinase; CaMKII = calcium/calmodulin-dependent protein kinase II; CSF = cerebrospinal fluid; AD = Alzheimer’s disease; BDNF-TrkB = brain-derived neurotrophic factor and its receptor; PHF-1 = paired helical filament-1 epitope of p-Tau; Aβ = amyloid-beta; APP = amyloid precursor protein; N/A = not applicable.

## Data Availability

The data are contained within the article.

## Abbreviations

AD: Alzheimer’s disease; Aβ, amyloid-beta; ACEA, arachidonyl-2-chloroethylamide; AHN, adult hippocampal neurogenesis; AKT/IRS1, protein kinase B/insulin receptor substrate 1; AMPA, α-amino-3-hydroxy-5-methyl-4-isoxazolepropionic acid receptor; AMPK, AMP-activated protein kinase; APP, amyloid precursor protein; APOE, apolipoprotein E; AT8, anti-phospho-Tau Ser202/Thr205 antibody; BACE-1, beta-site amyloid precursor protein cleaving enzyme 1; BDNF, brain-derived neurotrophic factor; CBD, cannabidiol; CB1, cannabinoid receptor type 1; CB2, cannabinoid receptor type 2; CDK5, cyclin-dependent kinase 5; CRMP, collapsin response mediator protein; CSF, cerebrospinal fluid; DAM, disease-associated microglia; DYRK1A, dual-specificity tyrosine-phosphorylation-regulated kinase 1A; ERAD, endoplasmic reticulum-associated degradation; Fyn, Fyn kinase; FTD, frontotemporal dementia; GSK-3β, glycogen synthase kinase 3 beta; GSH/GSSG, reduced/oxidized glutathione ratio; HEK293, human embryonic kidney 293 cells; HSPs, heat shock proteins; IFN-γ, interferon gamma; iNOS, inducible nitric oxide synthase; JNK, c-Jun N-terminal kinase; KEPI, kinase-enhanced PP1 inhibitor; LAMP1, lysosomal-associated membrane protein 1; MAPKs, mitogen-activated protein kinases; MAPT, microtubule-associated protein Tau; MDMA, 3,4-methylenedioxymethamphetamine; METH, methamphetamine; mTOR, mechanistic target of rapamycin; NFTs, neurofibrillary tangles; NMDA, N-methyl-D-aspartate; p-Tau, phosphorylated Tau; PC12, pheochromocytoma cells; PECO, population, exposure, comparison, outcome; PHF-1, paired helical filament-1; PI3K, phosphoinositide 3-kinase; PKC, protein kinase C; PP1, protein phosphatase 1; PP2A, protein phosphatase 2A; PSEN-1, presenilin-1; SH-SY5Y, human neuroblastoma cell line; Stx17, syntaxin 17; SUD, substance use disorder; Tau, microtubule-associated protein Tau; THC, delta-9-tetrahydrocannabinol; TREM2, triggering receptor expressed on myeloid cells 2; TrkB, tropomyosin receptor kinase B; WT, wild-type.
